# Residual-conditioned sparse transformer for photoacoustic image artifact reduction

**DOI:** 10.1016/j.pacs.2025.100731

**Published:** 2025-05-29

**Authors:** Xiaoxue Wang, Jinzhuang Xu, Chenglong Zhang, Moritz Wildgruber, Wenjing Jiang, Lili Wang, Xiaopeng Ma

**Affiliations:** a School of Control Science and Engineering, Shandong University, 250061, Jinan Shandong, China; b Department of Radiology, University Hospital, LMU Munich, D-81337, Munich, Germany; c Department of Obstetrics and Gynecology, Qilu Hospital of Shandong University, 250012, Jinan Shandong, China

**Keywords:** Photoacoustic tomography, Residual-conditioned sparse transformer, Sparse sampling, Artifact reduction

## Abstract

Photoacoustic tomography (PAT) combines the high spatial resolution of ultrasound imaging with the high contrast of optical imaging. To reduce acquisition time and lower the cost of photoacoustic imaging, sparse sampling strategy is often employed. Conventional reconstruction methods often produce artifacts when dealing with sparse data, affecting image quality and diagnostic accuracy. This paper proposes a Residual-Conditioned Sparse Transformer (RCST) network for reducing artifacts in photoacoustic images, aiming to enhance image quality under sparse sampling. By introducing residual prior information, our algorithm encodes and embeds it into local enhancement and detail recovery stages. We utilize sparse transformer blocks to identify and reduce artifacts while preserving key structures and details of the images. Experiments on multiple simulated and experimental datasets demonstrate that our method significantly suppresses artifacts and improves image quality, offering new possibilities for the application of photoacoustic imaging in biomedical research and clinical diagnostics.

## 1 Introduction

Photoacoustic tomography (PAT) is a promising medical imaging technique that combines the high spatial resolution of ultrasound imaging with the high contrast of optical imaging [1], [2], [3]. When a short pulse of laser light is delivered to a tissue, the absorbed energy causes a rapid thermal expansion, generating acoustic waves that can be detected by ultrasound transducers [4]. The spatial distribution of these acoustic signals is then used to reconstruct images of the tissue, revealing both structural and functional information. Photoacoustic imaging is extensively used in medical fields, such as tumor detection and characterization in oncology, blood flow and oxygen saturation assessment in cardiology, liver fibrosis evaluation in hepatology, and brain imaging and blood flow monitoring in neurology [5], [6], [7], [8], [9].

Multispectral optoacoustic tomography (MSOT) systems typically require hundreds of sensor elements to capture photoacoustic signals from multiple views to achieve high-resolution photoacoustic images. Optimizing the sensor array through hardware methods increases the complexity and cost of the system. Adopting sparse sampling strategy can meet the needs of data acquisition and processing, simplifying the design of the imaging system [10]. However, conventional reconstruction methods often rely on dense sampling and can struggle to produce high-quality images when faced with sparse data, leading to artifacts and inaccuracies (e.g., filtered back-projection algorithm, time reversal algorithm, model-based reconstruction algorithm) [11], [12], [13]. Compressed sensing is a revolutionary technique that exploits the sparsity of signals to reconstruct images [14]. Liu et al. developed the K-SVD dictionary learning technique to enhance the reconstruction in 3D PAT under sparse sampling [15]. It focuses on constructing a dictionary that captures the specific characteristics of the photoacoustic signals, leading to enhanced image fidelity. Furthermore, Tian et al. rotated the image clockwise and counterclockwise, averaging the results to cancel out the peaks and valleys of the stripes. It demonstrated effective stripe artifact removal in mouse abdominal imaging [16]. Nguyen et al. leveraged their spectral correlation with the original absorber characteristics and their signal appearance to identify and remove reflection artifacts in the images [17]. However, these methods rely on specific transducer array geometries and have limitations in terms of texture dependency, computational complexity, and adaptability to complex scenes.

Recently, deep learning has exhibited powerful capabilities in image processing and reconstruction. By learning features and patterns from large amounts of data, it can effectively identify and remove artifacts in PAT while preserving important details in the images [18], [19], [20]. In 2017, Antholzer et al. introduced CNN-based method for PAT, effectively mitigating artifacts and noise [21]. Daniel et al. demonstrated the effectiveness of U-Nets for reducing streak artifacts [22]. A 3D fully-dense U-net model eliminated the noise and artifacts by providing additional dense connections, as evidenced by successful restoration of blood vessels in human palm imaging [23]. Additionally, the application of generative models, such as generative adversarial networks and diffusion models facilitates the synthesis of high-quality images from sparse data [24]. Vu et al. utilized a Wasserstein generative adversarial network with gradient penalty (WGAN-GP) to effectively mitigate limited-view and limited-bandwidth artifacts in PAT [25]. Song et al. presented a diffusion model-based method for sparse-view reconstruction in PAT. It utilized learned prior information from data distribution as a constraint in the optimization framework. But it demands more iterations and substantial computational resources to generate high-quality images [26]. Supervised deep learning networks require a large amount of paired training data. To address this problem, Lu et al. introduced an unsupervised CycleGAN network and successfully achieved artifact removal [27]. An unsupervised artifact disentanglement network (PAT-ADN) was proposed to effectively remove artifacts under extremely sparse view conditions by disentangling artifacts and content components in the images [28]. However, these methods often lack the incorporation of specific prior information regarding image degradation, thereby failing to capture fine details and limiting their ability to process edge regions.

To address the limitations of conventional methods and deep learning algorithms in handling sparse data, we proposed a photoacoustic image artifact removal method based on Residual-Conditioned Sparse Transformer(RCST). This algorithm incorporates residual prior that contains specific information about image degradation. Then it encodes and embeds it into the stages of local enhancement and fine detail recovery. Furthermore, by integrating sparse transformer blocks, our method can identify and reduce artifacts while preserving the key structures and details of the images. The sparse attention mechanism focuses on important features in the images. Multi-scale information fusion ensures accurate recovery of details. Experiments on multiple simulated and experimental datasets have demonstrated the effectiveness of our method in terms of distortion measures and perceptual quality. It can significantly reduce artifacts and improve image quality under sparse sampling.

## 2 Methods

### 2.1 Residual-conditioned formulation for photoacoustics imaging

**Fig. 1:**
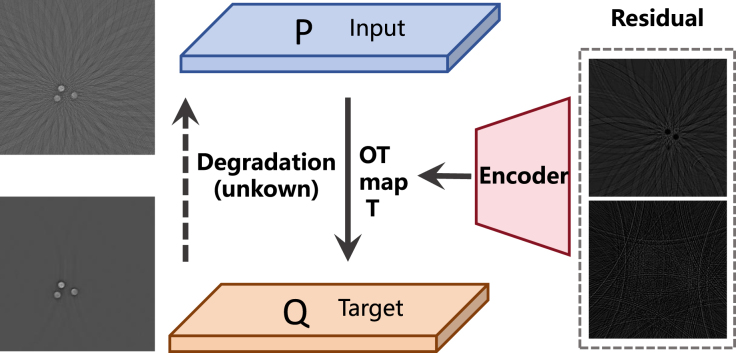
The core idea of Residual-Conditioned Sparse Transformer (RCST) network that uses residual as condition for photoacoustic imaging.

The image restoration is formulated as an Optimal Transport (OT) problem [29]. We denote the domains of degraded images and target images by Y and X, with their corresponding distributions P and Q, respectively. The optimal transport map is defined as the map T:Y→X that minimizes the transport cost among all possible transport maps (Fig. 1).

The Kantorovich [30] form of OT cost can be expressed as

(1) $$C_{kp}\left(P,Q\right)≜\underset{\pi \in \Pi \left(P,Q\right)}{\inf}\int_{Y\times X}c\left(y,x\right)d\pi \left(y,x\right).$$

Here, x represents the high-quality image, while y represents the degraded image. Taking the prior information of image degradation into account, we employ the Fourier Residual-guided Optimal Transport (FROT) objective. This involves incorporating a penalty term g(⋅) related to the degradation domain gap (i.e., the transport residual r=y−x) into the transport cost, resulting in $\overset{\sim }{c}\left(y,x\right)=c\left(x,y\right)+g\left(r\right)$. The FROT objective is defined as follows:

(2) $$\text{FROT}\left(P,Q\right)≜\underset{\pi \in \Pi \left(P,Q\right)}{\inf}\int_{X\times Y}\overset{\sim }{c}\left(y,x\right)d\pi \left(y,x\right).$$

The primary objective is to mitigate artifacts arising from sparse sampling in PAI. Then we introduce $l_{1}$ regularizer on Fourier residuals to address the artifacts. Specifically, the penalty function is defined as $g\left(\cdot \right)={\left\|F\left(\cdot \right)\right\|}_{1}$. Eq. (2) is formulated in the following dual form:

(3) $$\text{FROT}\left(P,Q\right)=\underset{\varphi }{\sup}\int_{Y}\varphi^{\overset{\sim }{c}}\left(y\right)dP\left(y\right)+\int_{X}\varphi \left(x\right)dQ\left(x\right).$$

The function $\varphi^{\overset{\sim }{c}}\left(y\right)\;=\;\inf_{x\in X}\left[c\left(x,y\right)+g\left(r\right)-\varphi \left(x\right)\right]$ represents the c-transform of φ. By applying the Rockafellar interchange theorem, we replace the optimization of the first term over the target x∈X with an equivalent optimization over the mapping T:Y→X. This substitution leads to the minimax reformulation of the dual form:

(4) $$\text{FROT}\left(P,Q\right)=\underset{\varphi }{\sup}\underset{T}{\inf}\left\{L\left(T,\varphi \right)≜\int_{X}\varphi \left(x\right)dQ\left(x\right)\right)\left(+\int_{Y}\left[c\left(T\left(y\right),y\right)+g\left(\overset{ˆ}{r}\left(T\right)\right)-\varphi \left(T\left(y\right)\right)\right]dP\left(y\right)\right\}.$$

The transport degradation domain gap, also known as the transport residual, is denoted by $\overset{ˆ}{r}\left(T\right)=y-T\left(y\right)$. Then we can approximate the mapping T and the potential φ through neural networks, specifically $T_{\theta }$ and $\varphi_{\omega }$ to address the minimax problem (4).

### 2.2 Residual-conditioned sparse transformer network

**Fig. 2:**
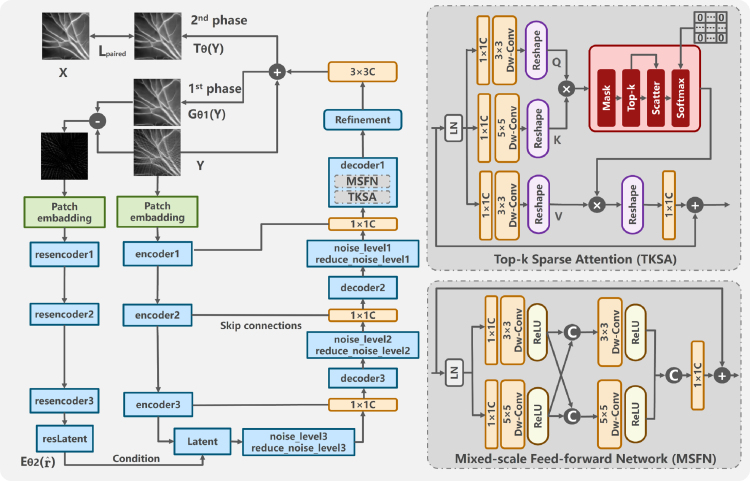
Overview of the proposed RCST network for photoacoustic image artifact reduction. RCST integrates top-k sparse attention (TKSA) and mixed-scale feed-forward network (MSFN).

As discussed in Section 2.1, the transport map is parameterized by a neural network $T_{\theta }:Y\to X$. To enhance the network’s ability to handle specific artifacts, RCST integrates the prior information of the degradation process into $T_{\theta }$ (Fig. 2). This network comprises an image generator $G_{\theta_{1}}$ and a residual encoder $E_{\theta_{2}}$, establishing the mapping from degraded images to high-quality images. The network includes two phases. The initial phase generates a preliminary restoration using $G_{\theta_{1}}$. Subsequently, the transport residual ${\overset{ˆ}{r}}_{0}=y-G_{\theta_{1}}\left(y\right)$ is determined, encapsulating the specific degradation information. The second phase involves encoding the degradation information with $E_{\theta_{2}}$ and integrating it into the subsequent refinement stage through a weighted combination mechanism. This specific mechanism allows the network to effectively utilize the residual information to enhance the refinement process. The transport residual functions as the conditional guide for local enhancement and detail recovery, thereby facilitating the identification and mitigation of artifacts arising from sparse sampling. Within the condition integration module, we adopt cross-stage feature fusion to amalgamate features from the image generator $G_{\theta_{1}}$ and the degradation-specific embedding $E_{\theta_{2}}\left({\overset{ˆ}{r}}_{0}\right)$, ensuring structurally coherent restoration [31]. This process incorporates top-k sparse attention (TKSA) and mixed-scale feed-forward network (MSFN) [32]. The transport process is described as follows:

(5) $${\overset{ˆ}{r}}_{0}=y-G_{\theta_{1}}\left(y\right),\;T_{\theta }\left(y\right)=G_{\theta_{1}}\left(y|E_{\theta_{2}}\left({\overset{ˆ}{r}}_{0}\right)\right).$$

Given the parameterization of $T_{\theta }$ and $\varphi_{\omega }$, the optimization objective function for Eq. (4) can be expressed as follows:

(6) $$L_{\mathrm{FROT}}\left(\omega ,\theta \right)=\;E_{x∼Q}\left[\varphi_{\omega }\left(x\right)\right]+$$

$$\;E_{y∼P}\left[c\left(T_{\theta }\left(y\right),y\right)+g\left(\overset{ˆ}{r}\left(T_{\theta }\right)\right)-\varphi_{\omega }\left(T_{\theta }\left(y\right)\right)\right].$$

Here, $T_{\theta }$ and $\varphi_{\omega }$ are the transport map and potential function, which are implemented by the generator and discriminator networks, respectively. The term $c\left(T_{\theta }\left(y\right),y\right)$ represents the cost function between the output image and the input degraded image, $g\left(r\left(T_{\theta }\right)\right)$ is the penalty term on the residuals, and $\varphi_{\omega }\left(T_{\theta }\left(y\right)\right)$ is the potential function evaluated at the transported image. The paired settings are utilized to ensure that $T_{\theta }\left(y\right)$ approximates the target x for each pair (y,x)∈M. M represents the set of paired subsets from X×Y. This is achieved using a squared $\ell_{2}$ loss function:

(7) $$L_{\text{paired}}\left(\theta \right)=\frac{\gamma }{\left|M\right|}\underset{\left(y,x\right)\in M}{\sum }{\left\|T_{\theta }\left(y\right)-x\right\|}^{2}.$$

Hence, the objective function for training is $\max_{\omega }\min_{\theta }\left\{L_{\text{FROT}}\left(\omega ,\theta \right)+L_{\text{paired}}\left(\theta \right)\right\}$. The overall loss function combines the FROT objective with a paired loss term to enhance the data fidelity and consistency in the training process.

Given an image $I\in R^{H\times W\times 3}$, H×W represents the spatial resolution of the feature map. We embed overlapping image patches through 3 × 3 convolution. In the network, we stack Sparse Transformer Blocks (STBs) to capture features that describe the spatially-varying artifacts distribution. Each encoder–decoder pipeline level is designed to handle specific spatial resolutions and channel dimensions, aiming to reveal the multi-scale representation of image degradation. We also utilize pixel-unshuffle and pixel-shuffle operations. In addition, we incorporate skip connections to bridge consecutive intermediate features. Within each STB, we replace the conventional self-attention mechanism with TKSA. It can enforce the sparsity of features and improve the efficiency of the feature aggregation process. Furthermore, we incorporate MSFN into the STBs. It enhances the representation of multi-scale local information, which is crucial for the image restoration process. This architecture enables the model to effectively enhance the quality of photoacoustic images that suffer from sparse sampling.

### 2.3 Sparse transformer blocks

The standard transformer employs all tokens in global self-attention computations, resulting in noisy interactions among unrelated features. The Sparse Transformer Blocks impose sparsity constraints on the attention mechanism. It not only enhances computational efficiency but also enables the capture of long-range dependencies within the data. Consequently, we integrate STBs as our feature extraction units. Specifically, for the input features at the (l-1)th block $X_{l-1}$, the encoding process can be formally expressed as:

(8) $$X_{l}^{\prime }=X_{l-1}+\text{TKSA}\left(\text{LN}\left(X_{l-1}\right)\right),$$

(9) $$X_{l}=X_{l}^{\prime }+\text{MSFN}\left(\text{LN}\left(X_{l}^{\prime }\right)\right),$$

where LN indicates the layer normalization. $X_{l}^{\prime }$ and $X_{l}$ represent the outputs of TKSA and MSFN, respectively.

**(a) Top-** k **sparse attention (TKSA)**

Given a query, key and value with dimensions $R^{L\times d}$, the output of the dot-product attention mechanism is typically expressed as:

(10) $$\text{Att}\left(Q,K,V\right)=\text{softmax}\left(\frac{QK^{⊤}}{\lambda }\right)V,$$

where Q, K, and V represent the matrix forms of the query, key, and value, respectively. The optional temperature factor λ is defined as $\lambda =\sqrt{d}$. Traditional multi-head self-attention mechanisms are based on densely fully-connected interactions, requiring the computation of attention scores between all queries and keys [33]. Each head processes subsets of Q, K, and V, and generates outputs with d=C/k channel dimensions. Then we apply the linear projection to obtain the final output. This approach can lead to excessive computational costs and introduce irrelevant feature interactions.

The sparse attention mechanism first encodes the channel-wise context through 1 × 1 convolutions. Subsequently, 3 × 3 depth-wise convolutions are employed to further extract local contextual features. Next, the similarities of pixel pairs between the reshaped queries and keys are calculated. These scores are stored in the transposed attention matrix M of size $R^{\overset{ˆ}{C}\times \overset{ˆ}{C}}$. According to the magnitude of the attention weights, the top-k largest weights are selected, and corresponding masks are generated. Only the elements within the range $\left[\Delta_{1},\Delta_{2}\right]$ from each row of M are normalized for softmax computation. For other elements lower than the top-k scores, we adopt the scatter function to set their probabilities to zero at the specified indices. The sparse attention weights enable the model to focus on the most important feature relationships. The mathematical expression is

(11) $$\text{SparseAtt}\left(Q,K,V\right)=\text{softmax}\left(T_{k}\left(\frac{QK^{⊤}}{\lambda }\right)\right)V,$$

where $T_{k}\left(\cdot \right)$ indicates the learnable top-k selection operator,

(12) $${\left[T_{k}\left(S\right)\right]}_{ij}=\left\{\begin{array}{cc} S_{ij}\; & S_{ij}\in \text{top-k(row}j\text{)}\\ 0\; & \text{otherwise.} \end{array}\right)$$

The output is combined with the value matrix through matrix multiplication. The outputs of all heads are concatenated, and the final result is produced through the linear projection.

**(b) Mixed-scale feed-forward network (MSFN)**

Single-scale depth-wise convolutions are typically introduced in feed-forward networks. It often overlooks the correlation between multi-scale artifacts. MSFN has been proven to have significant advantages in various image restoration tasks. It is designed to enhance feature extraction capabilities by effectively capturing multi-scale information from input feature maps. The input feature map is first expanded in the channel dimension through 1 × 1 convolution. Then the expanded feature map is processed through two parallel branches, each employing depth-wise convolutions of different kernel sizes: 3 × 3 and 5 × 5. These convolutions capture local features at different scales. The fusion process is achieved by concatenating the feature maps from both branches through another 1 × 1 convolution. The feature fusion process of MSFN can be formalized as:

(13) $${\overset{ˆ}{X}}_{l}=f_{1\times 1}^{c}\left(\text{LN}\left(X_{l-1}\right)\right),$$

$$X_{l}^{p_{1}}=\sigma \left(f_{3\times 3}^{dwc}\left({\overset{ˆ}{X}}_{l}\right)\right),\;X_{l}^{s_{1}}=\sigma \left(f_{5\times 5}^{dwc}\left({\overset{ˆ}{X}}_{l}\right)\right),$$

$$X_{l}^{p_{2}}=\sigma \left(f_{3\times 3}^{dwc}\left[X_{l}^{p_{1}},X_{l}^{s_{1}}\right]\right),\;X_{l}^{s_{2}}=\sigma \left(f_{5\times 5}^{dwc}\left[X_{l}^{s_{1}},X_{l}^{p_{1}}\right]\right),$$

$$X_{l}=f_{1\times 1}^{c}\left[X_{l}^{p_{2}},X_{l}^{s_{2}}\right]+X_{l-1},$$

where σ(⋅) denotes a ReLU activation function, and $f_{1\times 1}^{c}$ represents 1 × 1 convolution. Additionally, $f_{3\times 3}^{dwc}$ and $f_{5\times 5}^{dwc}$ refer to depth-wise convolutions with kernel sizes of 3 × 3 and 5 × 5, respectively. And [⋅] signifies a channel-wise concatenation.

## 3 Experiments

### 3.1 Datasets acquisition

The datasets include both simulated and experimental datasets. The simulated dataset was obtained from the retinal vasculature dataset DRIVE [34]. The experimental datasets were derived from publicly available datasets, including phantom and *in vivo* data [22].

**Fig. 3:**
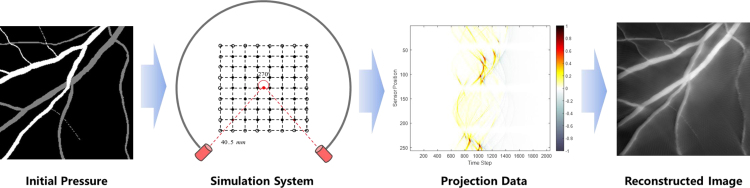
Flowchart for generating simulated vessel dataset via virtual PAT.

**Vessel dataset** The k-Wave [35] toolbox is employed to simulate forward pressure data to acquire adequate photoacoustic images at different sampling rates (32, 64, and 128 projections). The simulation settings are consistent with MSOT inVision 256 (iThera Medical GmbH, Neuherberg, Germany). This system consists of 256 piezoelectric transducers with the central frequency of 40 MHz. They cover the signal measurement range of 270 degrees and are placed on a circular arc with the radius of 45.5 mm. The speed of sound is set to 1530 m/s. The computational domain is divided into an 840 × 840 grid, with an absorbing layer of 10 grid points on each boundary. The imaging area, sized at 256 × 256 grid points, has pixel dimensions of 0.1 mm × 0.1 mm. The final simulated pressure signal comprises 2030 time points. We utilized the traditional back-projection algorithm to reconstruct the photoacoustic images. Fig. 3 illustrates the process of generating simulated vessel dataset via virtual PAT. It begins with the initial pressure image, which is then processed through a simulation system to model the structure and pressure distribution. The projection data, which represents the acoustic signals generated by light absorption in the vessels, is subsequently used to reconstruct image. The vessel dataset consists of 720 images, which were obtained through data augmentation.

**Phantom dataset and *in vivo* dataset** We utilized two experimental datasets: the phantom dataset comprising 469 images, and the *in vivo* dataset containing 274 abdominal images of mice. These images were acquired through full sampling (512 projections) and sparse sampling (32, 64, and 128 projections). We employed data augmentation to expand the test dataset to validate the effectiveness of our approach. For each sampling rate, the phantom dataset is partitioned into 449 images for training, 10 images for validation, and 30 images for testing, while the *in vivo* dataset is divided into 254 images for training, 10 images for validation, and 30 images for testing.

### 3.2 Experiment settings

The model consists of a generator Tnet and a discriminator Fnet for the task of photoacoustic image artifact removal. The structure of Tnet is shown in Fig. 2, and Fnet is a typical Convolutional Neural Network. The generator network aims to produce high-quality images that are visually indistinguishable from ground truth, while the discriminator network strives to accurately differentiate between generated images and ground truth. Through the adversarial training between the generator and discriminator, the model can learn richer feature representations, thereby enhancing the performance of image restoration. During the network training, we selected the RMSprop optimizer. RMSprop adapts the learning rate for each parameter based on the magnitude of the gradient. This adaptive learning rate mechanism helps in stabilizing the training process. The image size was set to 256 × 256, the batch size was set to 1, and the patch size of the training image was set to 128 × 128. The default learning rate was set to $1\times 10^{-4}$. Specifically, the learning rate for the generator Tnet was half of the default value, while the discriminator Fnet utilized the full default learning rate. This learning rate for Fnet decayed according to a preset momentum every 20 epochs. The decay strategy was implemented to gradually reduce the learning rate as the training progresses, which helps in fine-tuning the model parameters, achieving better convergence. The training process spanned 150 epochs, with model checkpoints saved every 10 epochs. Moreover, the performance of the model was monitored based on the validation set. The proposed method was implemented in PyTorch. The model was trained on the Ubuntu system equipped with an NVIDIA A100 GPU (40 GB) and 128 GB of RAM. We utilized Peak Signal-to-Noise Ratio (PSNR) and Structural Similarity Index Measure (SSIM) as the evaluation metrics [36].

## 4 Results

### 4.1 Vessel simulation results

**Fig. 4:**
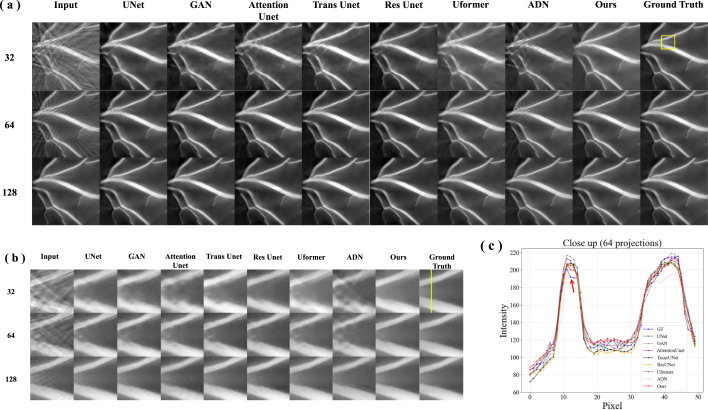
Vessel simulation results. (a) Results of artifact removal in PAT under different projections. The columns represent reconstruction results using UNet [22], GAN [37], Attention Unet [38], Trans Unet [39], Res Unet [40], Uformer [41], ADN [28], and our proposed algorithm, with rows indicating images reconstructed under 128, 64 and 32 projections. (b) The close-up images indicated by the yellow rectangle. (c) Intensity profiles along the yellow line in (b) under 64 projections.

To evaluate the performance of our proposed method, the reconstruction results based on UNet [22], GAN [37], Attention Unet [38], Trans Unet [39], Res Unet [40], Uformer [41], ADN [28] and our method were compared. Fig. 4(a) shows the simulated reconstruction results of vessel under different projections. The columns represent reconstruction results using different methods, with rows indicating images reconstructed under 32, 64 and 128 projections. It can be observed that the quality of the images decreases as the number of sampling points decreases, with more artifacts and less clarity. The comparative methods generally improve the image quality compared to the input, but the results still show some artifacts and lack fine details, especially under 32 and 64 projections. Uformer combines the strengths of transformers and UNets. It shows improved performance over standard UNet, with better detail preservation and less noise. Our method shows the best performance among all methods, with images that closely resemble the ground truth. It effectively removes artifacts and preserves details even under 32 projections.

The proposed method effectively manages the intricate structures and details inherent in the vessel images. The visual fidelity of the reconstructed images are further validated by their close resemblance to the ground truth, especially in the close-up images (Fig. 4(b)). Fig. 4(c) shows intensity profiles along the yellow line in Fig. 4(b) under 64 projections. The signal intensity of our method (red line) is remarkably close to that of the ground truth (blue line). The red arrow highlights that the recovery of signal peaks is particularly impressive under 64 projections, with both the positioning and intensity of the peaks closely matching the ground truth. The clarity of vessel edges and the contrast of internal structures are well-preserved. The signal curve generated by our proposed method is smooth, devoid of excessive fluctuations or noise. To further validate the effectiveness of our method, we performed quantitative analysis (Table 1). Our method achieves the highest SSIM values under different projections, indicating that it best preserves the contrast and structure of images. The SSIM values of ADN never exceed 0.90 under any condition, revealing that unsupervised methods face significant challenges when dealing with artifact removal in complex structural images. However, our method has a PSNR value of 25.340 dB, which is not the highest, yet it achieves the best visual quality (Fig. 4(a)(b)). Attention Unet and Trans Unet have PSNR values that do not exceed 32 dB, which is markedly lower than the 38.607 dB achieved by our proposed method under 128 projections.

Overall, our method effectively minimizes blur and artifacts compared to other comparative algorithms under different projections. It excels in retaining image details, particularly in the edges and texture regions, which are crucial for the accurate identification and analysis of vessels. This capability is essential for discerning subtle vessel structures from sparse data, thereby facilitating the detection of stenosis, blockages, or other abnormalities. In clinical applications, accurate reconstruction of vessel images can significantly aid doctors in better understanding the anatomical structure and pathological changes, thereby enabling more accurate diagnoses and treatment decisions.

**Table 1:** Comparison of quantitative results on simulated vessel dataset. **Bold** indicates the best results.

Metrics	PSNR			SSIM		
Datasets	Sparse32	Sparse64	Sparse128	Sparse32	Sparse64	Sparse128
UNet [22]	29.023	30.586	34.026	0.86789	0.91797	0.96116
GAN [37]	28.739	29.350	31.762	0.82548	0.86899	0.90962
Attention Unet [38]	28.475	28.340	30.111	0.80224	0.86472	0.90349
Trans Unet [39]	28.375	29.712	31.025	0.79425	0.88318	0.91928
Res Unet [40]	28.217	29.473	33.059	0.80411	0.88346	0.92714
Uformer [41]	28.987	31.454	36.402	0.85123	0.91593	0.94815
ADN [28]	**29.113**	30.331	30.693	0.81971	0.84856	0.88556
**Ours**	25.340	**32.104**	**38.607**	**0.89437**	**0.94104**	**0.96651**

### 4.2 Phantom validation

**Fig. 5:**
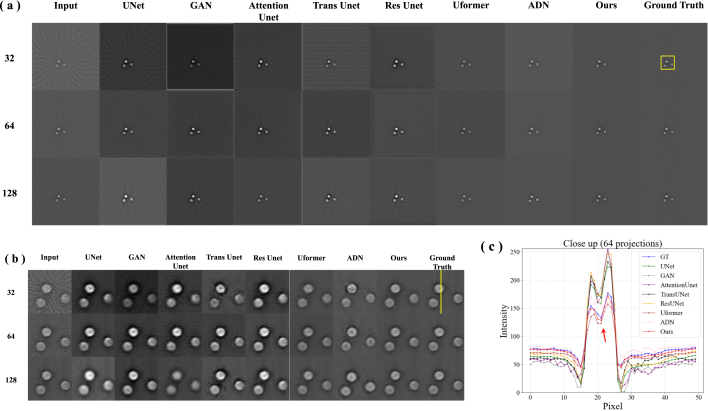
Phantom Validation. (a) Results of artifact removal in PAT under different projections. The columns represent reconstruction results using UNet [22], GAN [37], Attention Unet [38], Trans Unet [39], Res Unet [40], Uformer [41], ADN [28], and our proposed algorithm, with rows indicating images reconstructed under 128, 64 and 32 projections. (b) The close-up images indicated by the yellow rectangle. (c) Intensity profiles along the yellow line in (b) under 64 projections.

Fig. 5(a) shows the reconstruction results of different methods on the phantom dataset, including UNet [22], GAN [37], Attention Unet [38], Trans Unet [39], Res Unet [40], Uformer [41], ADN [28] and our proposed method. It can be observed that the reconstruction results of UNet and GAN exhibit some loss of detail and blurriness. The attention mechanism in Attention Unet assists the model in better focusing on important features, but the results are still blurry. Trans Unet performs better in capturing global information, but there is some loss of detail. Res Unet, Uformer and AND perform better in removing artifacts, but details and contrast still need to be improved. Upon closer examination of the close-up images (Fig. 5(b)), it becomes evident that these algorithms struggle with edge processing. The edges of the structures within the images appear less sharp and defined, which can significantly impact the overall image quality and the ability to discern fine details. Our proposed algorithm is able to better remove artifact under all conditions (32, 64 and 128 projections), and can clearly reconstruct the subtle structures in the image, such as edges and textures.

Fig. 5(c) illustrates intensity profiles along the yellow line in Fig. 5(b) under 64 projections. The results demonstrate our method (red line) is closest to the ground truth (blue line) in signal intensity. This indicates that it can more accurately capture the characteristics in the images. Moreover, our method performs excellently in the recovery of signal peaks, with the positioning and intensity of the peaks closely matching the ground truth. This demonstrates its superior ability to preserve edge details. The signal curve generated by our method is relatively smooth, without excessive fluctuations or noise, indicating that it has good stability. These comparative algorithms have deviations or fluctuations in signal strength in some areas. Table 2 shows the quantitative analysis of the performance of various methods. Our method achieves a PSNR value of 34.487 dB under 64 projections, outperforming the best-performing comparative method by approximately 9.2%. Our method demonstrates exceptional performance under different projections, with SSIM results consistently above 0.97. These results indicate that our algorithm has significant advantages in the accuracy of image reconstruction.

**Table 2:** Comparison of quantitative results on phantom dataset. **Bold** indicates the best results.

Metrics	PSNR			SSIM		
Datasets	Sparse 32	Sparse 64	Sparse 128	Sparse 32	Sparse 64	Sparse 128
UNet [22]	29.827	30.342	30.441	0.96933	0.97190	0.97426
GAN [37]	27.174	28.727	30.965	0.74300	0.84467	0.86798
Attention Unet [38]	28.821	31.587	31.104	0.70210	0.87899	0.90529
Trans Unet [39]	30.723	30.514	30.747	0.90894	0.90188	0.90461
Res Unet [40]	28.496	29.315	29.507	0.89992	0.95963	0.95429
Uformer [41]	26.488	29.677	30.462	0.92699	0.94172	0.97499
ADN [28]	29.151	30.245	30.352	0.94239	0.95372	0.94756
**Ours**	**32.903**	**34.487**	**34.209**	**0.97149**	**0.97883**	**0.98523**

### 4.3 In vivo validation

**Fig. 6:**
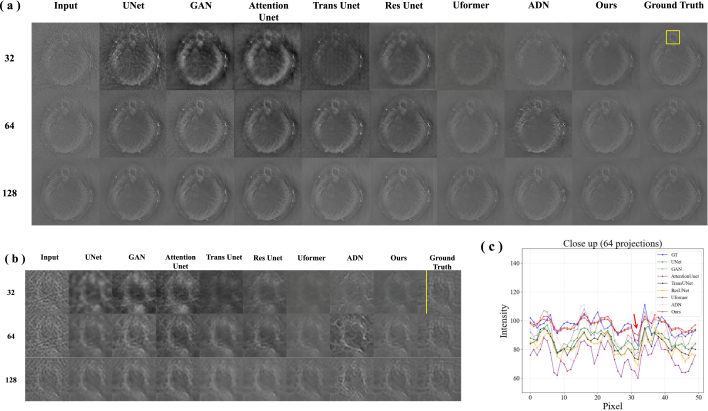
In vivo validation. (a) Results of artifact removal in PAT under different projections. The columns represent reconstruction results using UNet [22], GAN [37], Attention Unet [38], Trans Unet [39], Res Unet [40], Uformer [41], ADN [28], and our proposed algorithm, with rows indicating images reconstructed under 128, 64 and 32 projections. (b) The close-up images indicated by the yellow rectangle. (c) Intensity profiles along the yellow line in (b) under 64 projections.

To illustrate the practical utility of the proposed method, the reconstruction of mice’s abdomens using UNet [22], GAN [37], Attention Unet [38], Trans Unet [39], Res Unet [40], Uformer [41], ADN [28] and our proposed method were compared, respectively, as shown in Fig. 6. Due to sparse sampling in the spatial domain, the input images exhibit noticeable streak artifacts (Fig. 6(a)), which obscure the structural details and lead to a loss of image clarity. Although methods such as UNet, GAN, Attention Unet, Trans Unet, Res Unet, and ADN improve image quality to different extents compared to the input, they still suffer from artifacts and lack of fine detail. Our proposed method effectively mitigates artifacts under sparse sampling, resulting in clearer details, as evidenced by the close-up images in Fig. 6(b). However, Uformer and our proposed method both tend to over-smooth the image reconstructions under 32 projections. This issue is presumably attributable to the intricate stripe-like details present in the images. Fig. 6(c) displays intensity profiles along yellow line under 64 projections, further validating the superior performance of our algorithm in signal strength recovery, closely aligning with the ground truth, as highlighted by the red arrows.

In the analysis of the experimental results on *in vivo* dataset, it is evident that our proposed method outperforms other comparative methods in terms of PSNR and SSIM (Table 3). Specifically, our algorithm achieves the highest PSNR values under all sparse sampling conditions, with a PSNR of 32.687 dB under 128 projections, which is approximately 8.9% higher than the second-best method. In terms of SSIM, our method also demonstrates superior performance, achieving values of 0.88714, 0.90494, and 0.93217 under 32, 64, and 128 projections, respectively, all of which are the highest among the methods. These findings validate that it provides higher quality reconstruction results. This advantage stems from the algorithm’s optimization in feature extraction, making it more effective in processing complex images.

**Table 3:** Comparison of quantitative results on *in vivo* dataset. **Bold** indicates the best results.

Metrics	PSNR			SSIM		
Datasets	Sparse 32	Sparse 64	Sparse 128	Sparse 32	Sparse 64	Sparse 128
UNet [22]	27.268	28.739	30.015	0.74398	0.88674	0.90290
GAN [37]	28.549	28.487	28.225	0.55404	0.61655	0.72214
Attention Unet [38]	28.396	27.591	28.770	0.61861	0.72829	0.75774
Trans Unet [39]	28.434	28.181	28.679	0.57511	0.72304	0.80760
Res Unet [40]	27.379	28.545	28.455	0.76432	0.79879	0.86420
Uformer [41]	26.107	27.514	29.710	0.87212	0.86719	0.90262
ADN [28]	28.849	28.393	28.726	0.70064	0.72718	0.74676
**Ours**	**29.978**	**30.584**	**32.687**	**0.88714**	**0.90494**	**0.93217**

### 4.4 Ablation study

We conducted ablation experiments to verify the effectiveness of the residual module, TKSA, and MSFN. The results are shown in Table 4. The ablation experiments were conducted on the phantom dataset using different combinations of modules for validation. The results show a progressive enhancement in model performance with the incorporation of each module. Our proposed method consistently achieves the highest PSNR values under all conditions, demonstrating its superior performance. When all three modules are integrated, they collectively empower the network to adeptly handle intricate features and data variability, thereby enhancing its robustness and adaptability.

**Table 4:** Ablation study results on phantom dataset (PSNR (dB)). **Bold** indicates the best results.

Residual	MSFN	TKSA	Sparse 32	Sparse 64	Sparse 128
–	–	–	31.626	34.361	33.837
✓	–	–	31.466	34.413	34.157
✓	✓	–	32.343	34.407	34.185
✓	✓	✓	**32.903**	**34.487**	**34.209**

## 5 Discussion and conclusion

PAT has demonstrated significant potential in early diagnosis and treatment planning. Sparse sampling strategies are often employed to simplify the systems and lower the cost. Traditional reconstruction methods often produce artifacts when dealing with sparse data. This paper introduces the Residual-Conditioned Sparse Transformer (RCST) network to significantly improve artifact reduction and detail recovery. The RCST network incorporates residual prior information and encodes it into local enhancement and detail recovery stages. By integrating sparse transformer blocks, the network can focus on important features in the images, and reduce artifacts while preserving key structures and details. Quantitative analysis was conducted to verify the superiority of our proposed method. Our method achieved the highest SSIM values under different projections in the vessel dataset, indicating its superior ability to preserve image contrast and structure. In the phantom dataset, our proposed method achieved a PSNR value that is approximately 9.2% higher than the best-performing comparative method under 64 projections. Similarly, our method consistently outperformed other methods under all sparse sampling conditions on *in vivo* dataset, with improvements of around 8.9% over the second-best method under 128 projections. Experimental results on multiple simulated and experimental datasets have provided strong evidence of the superior performance of our proposed method.

This study contributes to the development of more efficient and cost-effective imaging solutions. It incorporates residual prior information and integrates sparse transformer blocks, enabling adaptive attention on salient features while mitigating irrelevant interactions. This architecture enhances feature extraction effectiveness, thereby improving image quality under sparse sampling conditions. However, the reliance on supervised learning necessitates a substantial amount of paired training data. Acquiring such data is particularly challenging and time-consuming, where high-quality ground truth images may not always be readily available. Future research could further investigate sparse sampling reconstruction methods in PAT, including developing semi-supervised or unsupervised learning approaches, refining the training process, and enhancing computational efficiency. Validating these deep learning approaches is necessary, ensuring they are capable of handling diverse clinical and research applications.

## CRediT authorship contribution statement

**Xiaoxue Wang:** Writing – review & editing, Writing – original draft, Validation, Methodology. **Jinzhuang Xu:** Writing – review & editing, Writing – original draft. **Chenglong Zhang:** Writing – review & editing. **Moritz Wildgruber:** Writing – review & editing. **Wenjing Jiang:** Writing – review & editing. **Lili Wang:** Writing – review & editing. **Xiaopeng Ma:** Writing – review & editing.

## Declaration of competing interest

The authors declare that they have no known competing financial interests or personal relationships that could have appeared to influence the work reported in this paper.

## Data Availability

Data will be made available on request.

## References

[1] Wang L.V., Hu S. (2012). Photoacoustic tomography: In Vivo imaging from organelles to organs. Science.

[2] Wang L. (2009). Multiscale photoacoustic microscopy and computed tomography. Nat. Photonics.

[3] Tian C., Zhang C., Zhang H., Xie D., Jin Y. (2021). Spatial resolution in photoacoustic computed tomography. Rep. Progr. Phys..

[4] Xia J., Yao J., Wang L.V. (2014). Photoacoustic tomography: principles and advances. Electromagn. Waves (Camb. Mass.).

[5] Farajollahi A., Baharvand M. (2024). Advancements in photoacoustic imaging for cancer diagnosis and treatment. Int. J. Pharm..

[6] Liu S., Zhang R., Han T., Pan Y., Zhang G., Long X., Zhao C., Wang M., Li X., Yang F., Sang Y., Zhu L., He X., Li J., Zhang Y., li C., Jiang Y., Yang M. (2022). Validation of photoacoustic/ultrasound dual imaging in evaluating blood oxygen saturation. Biomed. Opt. Express.

[7] Menozzi L., Del Águila Á., Vu T., Ma C., Yang W., Yao J. (2023). Three-dimensional non-invasive brain imaging of ischemic stroke by integrated photoacoustic, ultrasound and angiographic tomography (PAUSAT). Photoacoustics.

[8] Li W., Lv J., Li H., Song L., Zhang R., Zhao X., Xuan F., Sun T., Long K., Zhao Y. (2025). Quantification of vascular remodeling and sinusoidal capillarization to assess liver fibrosis with photoacoustic imaging. Radiology.

[9] Qiu Y., Li H., Yu K., Chen J., Qi L., Zhao Y., Nie L. (2025). Collagen fibers quantification for liver fibrosis assessment using linear dichroism photoacoustic microscopy. Photoacoustics.

[10] Li X., Zhang S., Wu J., Huang S., Feng Q., Qi L., Chen W. (2020). Multispectral interlaced sparse sampling photoacoustic tomography. IEEE Trans. Med. Imaging.

[11] Wei J., Shen K., Tian C., Zhang Z., Qu J., Li B. (2023). Second Conference on Biomedical Photonics and Cross-Fusion.

[12] Biton S., Arbel N., Drozdov G., Gilboa G., Rosenthal A. (2019). Optoacoustic model-based inversion using anisotropic adaptive total-variation regularization. Photoacoustics.

[13] Zhu J., Huynh N., Ogunlade O., Ansari R., Lucka F., Cox B., Beard P. (2023). Mitigating the limited view problem in photoacoustic tomography for a planar detection geometry by regularized iterative reconstruction. IEEE Trans. Med. Imaging.

[14] Zhang X., Ma F., Zhang Y., Wang J., Liu C., Meng J. (2022). Sparse-sampling photoacoustic computed tomography: Deep learning vs. compressed sensing. Biomed. Signal Process. Control..

[15] Liu F., Gong X., Wang L.V., Guan J., Song L., Meng J. (2019). Dictionary learning sparse-sampling reconstruction method for in-vivo 3D photoacoustic computed tomography. Biomed. Opt. Express.

[16] Dong W., Zhu C., Xie D., Zhang Y., Tao S., Tian C. (2024). Image restoration for ring-array photoacoustic tomography system based on blind spatially rotational deconvolution. Photoacoustics.

[17] Nguyen H., Hussain A., Steenbergen W. (2018). Reflection artifact identification in photoacoustic imaging using multiwavelength excitation. Biomed. Opt. Express.

[18] Gröhl J., Schellenberg M., Dreher K., Maier-Hein L. (2021). Deep learning for biomedical photoacoustic imaging: A review. Photoacoustics.

[19] Tian C., Shen K., Dong W., Gao F., Wang K., Li J., Liu S., Feng T., Liu C., Li C., Yang M., Wang S., Tian J. (2024). Image reconstruction from photoacoustic projections. Photonics Insights.

[20] Liu C., Gao R., Cheng J., Wang S., Hou W., Dong S., Wang Y., Fu X., Zeng S., Yaguang R., Ma X., Liu J., Sun M. (2024).

[21] Antholzer S., Haltmeier M., Schwab J. (2017). Deep learning for photoacoustic tomography from sparse data. Inverse Probl. Sci. Eng..

[22] Davoudi N., Dean-Ben X.L., Razansky D. (2019). Deep learning optoacoustic tomography with sparse data. Nat. Mach. Intell..

[23] Zheng W., Zhang H., Huang C., Shijo V., Xu C., Xu W., Xia J. (2023). Deep learning enhanced volumetric photoacoustic imaging of vasculature in human. Adv. Sci..

[24] Arjovsky M., Chintala S., Bottou L., Precup D., Teh Y.W. (2017). Proceedings of the 34th International Conference on Machine Learning.

[25] Vu T., Li M., Humayun H., Zhou Y., Yao J. (2020). A generative adversarial network for artifact removal in photoacoustic computed tomography with a linear-array transducer. Exp. Biol. Med..

[26] Song X., Wang G., Zhong W., Guo K., Li Z., Liu X., Dong J., Liu Q. (2023). Sparse-view reconstruction for photoacoustic tomography combining diffusion model with model-based iteration. Photoacoustics.

[27] Lu M., Liu X., Liu C., Li B., Gu W., Jiang J., Ta D. (2021). Artifact removal in photoacoustic tomography with an unsupervised method. Biomed. Opt. Express.

[28] Zhong W., Li T., Hou S., Zhang H., Li Z., Wang G., Liu Q., Song X. (2024). Unsupervised disentanglement strategy for mitigating artifact in photoacoustic tomography under extremely sparse view. Photoacoustics.

[29] Tang X., Hu X., Gu X., Sun J. (2024). Proceedings of the 41st International Conference on Machine Learning.

[30] Kantorovich L. (2006). On the translocation of masses. J. Math. Sci..

[31] S.W. Zamir, A. Arora, S. Khan, M. Hayat, F.S. Khan, M.-H. Yang, L. Shao, Multi-Stage Progressive Image Restoration, in: 2021 IEEE/CVF Conference on Computer Vision and Pattern Recognition, CVPR, 2021, pp. 14816–14826.

[32] X. Chen, H. Li, M. Li, J. Pan, Learning A Sparse Transformer Network for Effective Image Deraining, in: 2023 IEEE/CVF Conference on Computer Vision and Pattern Recognition, CVPR, 2023, pp. 5896–5905.

[33] S.W. Zamir, A. Arora, S. Khan, M. Hayat, F.S. Khan, M.-H. Yang, Restormer: Efficient Transformer for High-Resolution Image Restoration, in: 2022 IEEE/CVF Conference on Computer Vision and Pattern Recognition, CVPR, 2022, pp. 5718–5729.

[34] Staal J., Abramoff M., Niemeijer M., Viergever M., van Ginneken B. (2004). Ridge-based vessel segmentation in color images of the retina. IEEE Trans. Med. Imaging.

[35] Treeby B.E., Cox B.T. (2010). K-wave: MATLAB toolbox for the simulation and reconstruction of photoacoustic wave fields. J. Biomed. Opt..

[36] A. Horé, D. Ziou, Image Quality Metrics: PSNR vs. SSIM, in: 2010 20th International Conference on Pattern Recognition, 2010, pp. 2366–2369.

[37] P. Isola, J.-Y. Zhu, T. Zhou, A.A. Efros, Image-to-Image Translation with Conditional Adversarial Networks, in: 2017 IEEE Conference on Computer Vision and Pattern Recognition, CVPR, 2017, pp. 5967–5976.

[38] Oktay O., Schlemper J., Folgoc L.L., Lee M.J., Heinrich M.P., Misawa K., Mori K., McDonagh S.G., Hammerla N.Y., Kainz B., Glocker B., Rueckert D. (2018). http://arxiv.org/abs/1804.03999.

[39] Chen J., Lu Y., Yu Q., Luo X., Adeli E., Wang Y., Lu L., Yuille A., Zhou Y. (2021).

[40] K. He, X. Zhang, S. Ren, J. Sun, Deep Residual Learning for Image Recognition, in: 2016 IEEE Conference on Computer Vision and Pattern Recognition, CVPR, 2016, pp. 770–778.

[41] Z. Wang, X. Cun, J. Bao, W. Zhou, J. Liu, H. Li, Uformer: A General U-Shaped Transformer for Image Restoration, in: 2022 IEEE/CVF Conference on Computer Vision and Pattern Recognition, CVPR, 2022, pp. 17662–17672.

